# Evolution of 14-3-3 Proteins in Angiosperm Plants: Recurring Gene Duplication and Loss

**DOI:** 10.3390/plants10122724

**Published:** 2021-12-11

**Authors:** Yulia V. Mikhaylova, Roman K. Puzanskiy, Maria F. Shishova

**Affiliations:** 1 Laboratory of Biosystematics and Cytology, Komarov Botanical Institute of the Russian Academy of Sciences, Professor Popov str., 2, 197376 St. Petersburg, Russia; 2 Laboratory of Analytical Phytochemistry, Komarov Botanical Institute of the Russian Academy of Sciences, Professor Popov str., 2, 197376 St. Petersburg, Russia; puzansky@yandex.ru; 3 Department of Plant Physiology and Biochemistry, Saint-Petersburg State University, Universitetskaya em., 7/9, 199034 St. Petersburg, Russia

**Keywords:** 14-3-3 proteins, whole-genome duplication, coexpression, gene family, molecular phylogeny, MrBayes

## Abstract

14-3-3 proteins are key regulatory factors in plants and are involved in a broad range of physiological processes. We addressed the evolutionary history of 14-3-3s from 46 angiosperm species, including basal angiosperm *Amborella* and major lineage of monocotyledons and eudicotyledons. Orthologs of *Arabidopsis* isoforms were detected. There were several rounds of duplication events in the evolutionary history of the 14-3-3 protein family in plants. At least four subfamilies (iota, epsilon, kappa, and psi) formed as a result of ancient duplication in a common ancestor of angiosperm plants. Recent duplication events followed by gene loss in plant lineage, among others Brassicaceae, Fabaceae, and Poaceae, further shaped the high diversity of 14-3-3 isoforms in plants. Coexpression data showed that 14-3-3 proteins formed different functional groups in different species. In some species, evolutionarily related groups of 14-3-3 proteins had coexpressed together under certain physiological conditions, whereas in other species, closely related isoforms expressed in the opposite manner. A possible explanation is that gene duplication and loss is accompanied by functional plasticity of 14-3-3 proteins.

## 1. Introduction

The 14-3-3 (or GF14) proteins belong to the conservative family of proteins, present in all eukaryotes. 14-3-3s modulate activity of various targets in a cell via protein–protein interaction in phosphorylation-dependent mode [1]. The first protein from the family was discovered in a cow brain in 1967 [2]. 25 years later, 14-3-3s almost simultaneously were found in plants: *Arabidopsis thaliana* [3], maize *Zea mays* [4], spinach *Spinacea aleracea*, pea *Pisum* and herbaceous plant *Oenothera* [5]. In plants, 14-3-3 family members are involved in regulation of processes such as primary metabolism, cell division, growth and differentiation, light, hormone and stress signalling, as well as plant innate immunity [6,7,8,9,10]. Notably, the 14-3-3 proteins do not have their own enzymatic activity. Its mechanism of action corresponds with a facility to perform physical protein–protein interactions. Intensive investigations revealed more than 300 potential targets of 14-3-3 proteins [8]. The 14-3-3 proteins interact with each other and form homo- or hetero-dimers [11]. Thus, dimers have two domains for interacting with target proteins, which are assumed to be phosphorylated in most cases at the amino acid residues of serine or threonine [12,13].

Green plants have the highest number of 14-3-3 isoforms. Protists and fungi have from one to four isoforms [14]. In mammals, seven 14-3-3 isoforms were discovered and named by Greek letters (beta/alpha, gamma/delta, epsilon, etc.) [1]. In plants, isoforms were denoted firstly also by Greek letters, but starting from the end of the alphabet: omega, psi, chi, phi, etc. Alternatively, 14-3-3s in plants are known as General Regulatory Factors (GRF), with an index number. The total number of 14-3-3 isoforms is different in various plant species. There are 13 isoforms in *Arabidopsis thaliana* [15], 17 isoforms in tobacco *Nicotiana tabacum* [16], 25 in banana *Musa acuminate* [17] and 7 in a model grass *Brachypodium distachyon* [18].

The large number of isoforms suggests a very high combinatorial complexity in dimer arrangement, which fits a regulatory function of the 14-3-3 proteins [19]. Interaction of target proteins with 14-3-3 proteins apparently alters its stability and cell localization. The effect might be revealed at all levels from transcriptional to post-translational. Elevation of system plasticity also depends on the fact that 14-3-3s themselves are affected, both transcriptionally and functionally, by the internal and external factors. Accumulated information about 14-3-3 proteins might indicate both the specificity and redundancy of the regulatory net. Unfortunately, we still have a very limited knowledge on relative affinities of different isoforms toward each single 14-3-3 target.

One of the first phylogenetic analyses, which focused its attention on plant 14-3-3 proteins, revealed five evolutionary groups [20]: omega group, psi group, epsilon group, kappa subfamily, and separate groups of monocots with very low statistical support. A more comprehensive study of 14-3-3 protein evolution includes 125 protein sequences from 48 eukaryotic organisms [14], and confirms the division of plant 14-3-3s into four groups of subfamilies (omega, kappa, psi and epsilon), but monocots cluster together with the psi group. Further research supports the principal separation of epsilon and non-epsilon evolutionary groups [17,18,21].

The number of revealed 14-3-3 sequences in plants has increased both in terms of isoforms and species, and the phylogeny needs to be updated. The most comprehensive phylogenetic study was based on 14-3-3 sequences from 21 seed plants [14]. In recent decades, next-generation sequencing techniques have allowed us to obtain genetic data for several dozens of plant species. Many new isoforms of 14-3-3 proteins have been characterized in various plant species, for instance in banana [17], poplar [22], apple [23], common bean [24], etc.

In the present work, we performed evolutionary analysis of plant 14-3-3 proteins in a broad phylogenetic scale. We analysed 14-3-3 proteins in flowering plants including not only well-studied model plants, but also those important for understanding of angiosperm evolution species, such as ancient basal angiosperm *Amborella*. We found in analysed species orthologs of *Arabidopsis* 14-3-3 proteins subfamilies, and checked whether evolutionarily close isoforms had functional similarity.

## 2. Results

### 2.1. Phylogenetic Analysis of 14-3-3 Proteins Family 

To study evolution of plant 14-3-3 proteins, we sampled 46 plant species from different branches of the angiosperm phylogenetic tree [25]. *Amborella trichopoda* is a plant from the basal angiosperms, which had arisen before diversification of monocotyledons and eudicotyledons. We sampled 13 monocot species, one from order Zingberales (*Musa acuminata*–banana), and 12 from order Poales. Eleven Poales species were from the grasses family (Poaceae) and one from Bromeliaceae (*Ananas*). In total, 32 species were from Eudicots: basal eudicots (*Aquilegia*), basal Superasterids group (*Amaranthus*), Lamiids (Lamiales–1, Solanales–3), Campanulids (*Daucus carota*), Fabids (Malpighiales–5, Fabales–4, Rosales–1, Cucurbitales–1), Malvids (Malvales–2, Myrtales–2, Sapindales–2, Brassicales–10). Multiple alignment made for the phylogenetic tree reconstruction is presented in Supplementary Dataset S4. 

We used two approaches for phylogenetic analysis, neighbour-joining (NJ) and Bayesian inference. There were more clades with good statistical support in the Bayesian tree than in the NJ tree. Neighbour-joining is a distance-based method, whereas the Bayesian approach is a more complicated method that allows the implementation of complex evolution models. The Bayesian method seems to be best choice for phylogenetic analysis of 14-3-3 proteins, whereas NJ phylogenetic trees often have many clades with extremely low statistical support (bootstrap values < 50), and allow us to discriminate only epsilon and non-epsilon isoforms (for example [17]). With the benefit of Bayesian analysis, we distinguished subfamilies of 14-3-3 proteins inside the epsilon and non-epsilon group. Three transcripts from rice (Os07g28430, Os11g35030, Os10g30880) and one from wheat (C424EC0B1) had such few homological regions with other 14-3-3 proteins that it was not possible to locate them in a particular clade, therefore they were excluded.

**Figure 1:**
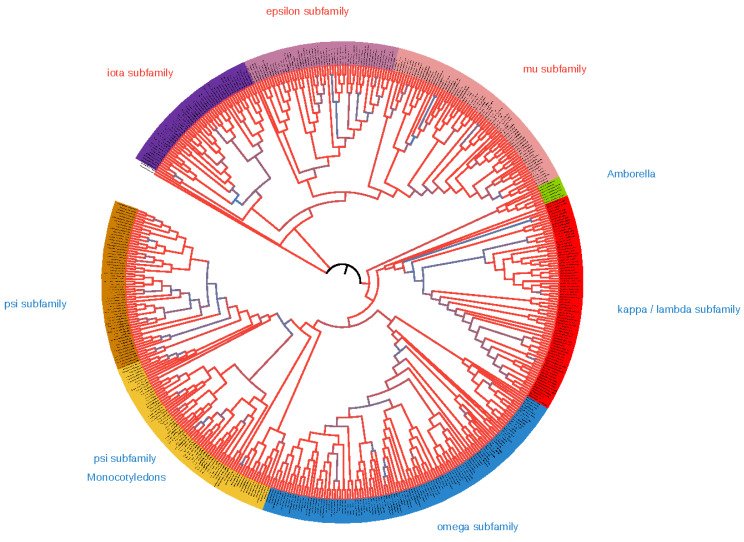
Phylogenetic tree (cladogram) of plant 14-3-3 proteins family based on Bayesian analysis of coding nucleotide sequences. Branches are coloured according to Bayesian support values, maximal support is red, minimal support is blue. Section colours indicate subfamilies. Text colours indicate epsilon (red) and non-epsilon (blue) types of isoforms. Tips of branches are omitted. Full phylogenetic tree in high resolution with all analysed 14-3-3 proteins names is presented in Supplementary (Figure S1).

**Figure 2:**
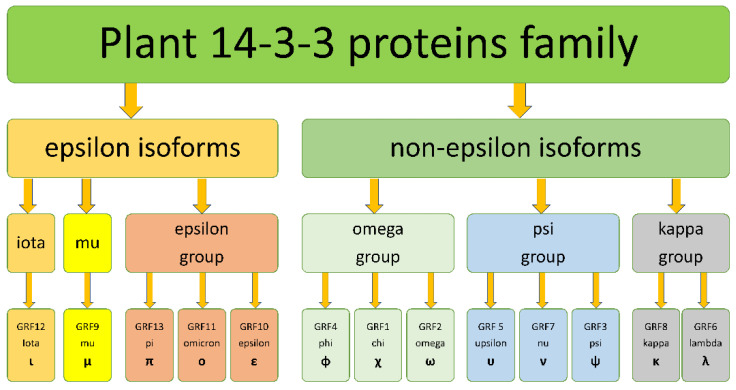
*Arabidopsis*-based hierarchic classification of plant 14-3-3 proteins subfamilies. All plant 14-3-3s subdivided into two types of isoforms: epsilon and non-epsilon. At the next level of hierarchy there are six subfamilies: iota, mu, epsilon, omega, psi and kappa. In *Arabidopsis,* most of subfamilies include several isoforms, named by Greek letters or GRFs (General Regulatory Factors).

The total number of 511 plant sequences broke into two major clades with high (0.99–1) support (Figure 1 and Figure S1), 163 in an epsilon clade and 292 in a non-epsilon clade. There were three subclades in the epsilon clade: mu (74), iota (51) and epsilon groups (55), including epsilon itself, omicron and pi isoforms. The mu subfamily was a sister to the epsilon subfamily, whereas iota had a basal position in the epsilon clade. Non-epsilon isoforms included three groups: omega (114), psi (117) and kappa (77). The omega group included phi, chi and omega; the psi group included upsilon, nu and psi; the kappa group included kappa and lambda isoforms. Hierarchic classification of subfamilies is summarized in Figure 2. All main groups of isoforms formed on the phylogenetic tree clades with high values of posterior probability. One rice isoform (Os.01g11110) was in the epsilon clade, but did not cluster inside one of the subfamily clades. Six *Amborella*’s and one wheat isoform joined together in a well-supported clade, which took a basal place in the non-epsilon clade.

Subfamilies of 14-3-3 proteins were distributed between plant species in an uneven manner. Almost all sampled Eudicots as well as *Amborella* had isoforms from the iota subfamily, whereas amongst monocots only banana had one iota 14-3-3 protein. There were no iota isoforms in pineapple. Grasses had only the epsilon isoform itself and had neither iota, nor mu isoforms. Some dicot species had several paralogous isoforms from iota and mu subfamilies; among these are apple, soybean, poplar, willow, flax, and aquilegia (Table S3). Epsilon isoforms were absent in species from the Fabaceae family: beans and medic. Isoforms pi, omicron and epsilon are paralogs belonging to *A. thaliana*. It was possible to determine orthologous genes for each of three these isoforms only in Brassicaceae, whereas in other taxonomic groups we should imply a joined epsilon–omicron–pi subfamily, or epsilon group. The same diversification pattern was observed for non-epsilon types of isoforms: omega group, psi group and kappa group. The omega group was the most widespread subfamily of plant 14-3-3 proteins. We did not find omega-group isoforms only in banana and *Amborella*. The majority of species had several duplicated isoforms from the omega subfamily. The psi group was the biggest subfamily of 14-3-3s, particularly many isoforms from the psi group were found in Poales (56 isoforms in 12 species). Seven isoforms were found in wheat and maize, five in each foxtail species. Kappa/lambda isoforms were present in all species except grasses. 

Based on phylogenetic relationship, we allocated 14-3-3 isoforms among the main subfamilies (Table S3). Separations of phi, chi and omega in the omega group, upsilon, nu and psi in psi group, kappa and lambda in kappa group, took place only in the Brassicaceae lineage. Thus, we can use these Greek names for other *Arabidopsis* species, *A. lyrate* and *A. helleri*, as well as for other Brassicaceae, but not for other plant lineages. For instance, plants from the Solanaceae family (tomato, potato, tobacco) have at least four isoforms in the omega group. Diversification of these four isoforms in the Solanaceae lineage and diversification of three (omega, phi and chi) isoforms in *Arabidopsis* were independent events. For convenience, this lineage-specific ortholog we denoted by Latin letters, so the omega group in Solanaceae includes omega A, omega B, omega C and omega D isoforms. The most recent isoforms, which exist as paralogous in particular species, we denoted by number. For instance, *Nicotiana tabacum* has two paralogous omega D isoforms, which we named omega D.1 and omega D.2. This system of indexes reflects the evolutionary origin of 14-3-3 diversity. 

### 2.2. Coexpression of 14-3-3 Protein Isoforms in Plants

**Figure 3:**
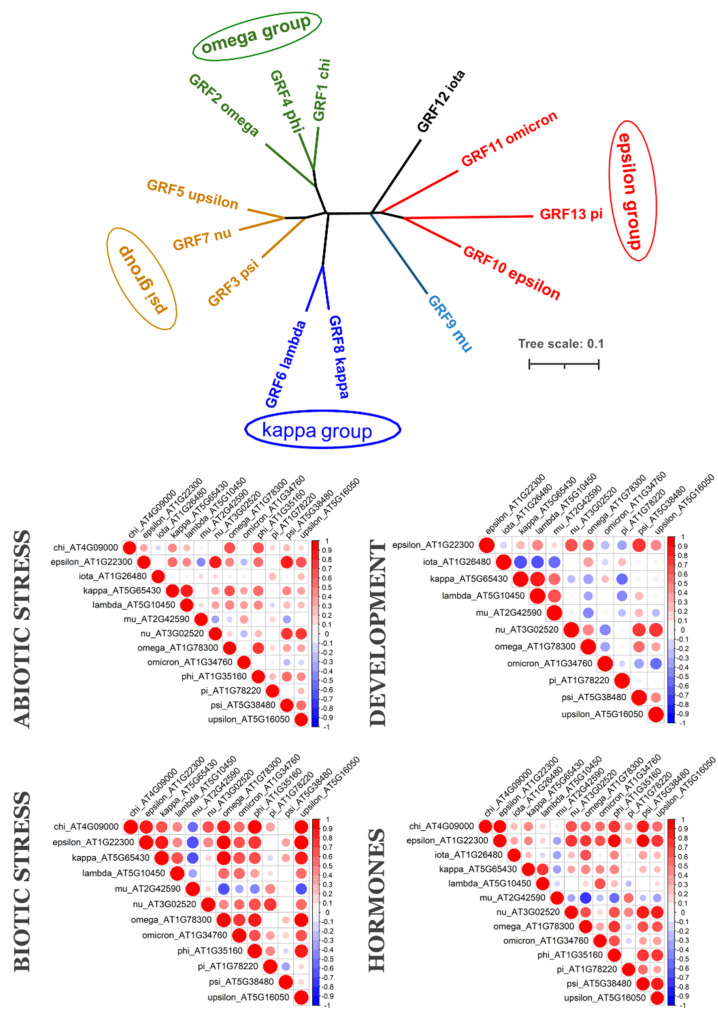
*Arabidopsis thaliana* 14-3-3 isoforms: phylogenetic relationships (upper graph) and coexpression under different physiological conditions (correlation heatmaps). Upper graph is unrooted NJ tree of 14-3-3s from *A. thaliana*, bar is number of nucleotide changes per site. Colour of a branch reflects different subfamilies groups. Correlation heatmaps show PCCs between each pair of 14-3-3s. Positive correlations are displayed in red, negative correlations are displayed in blue. The size of the circle and the colour intensity are proportional to the PCCs.

**Figure 4:**
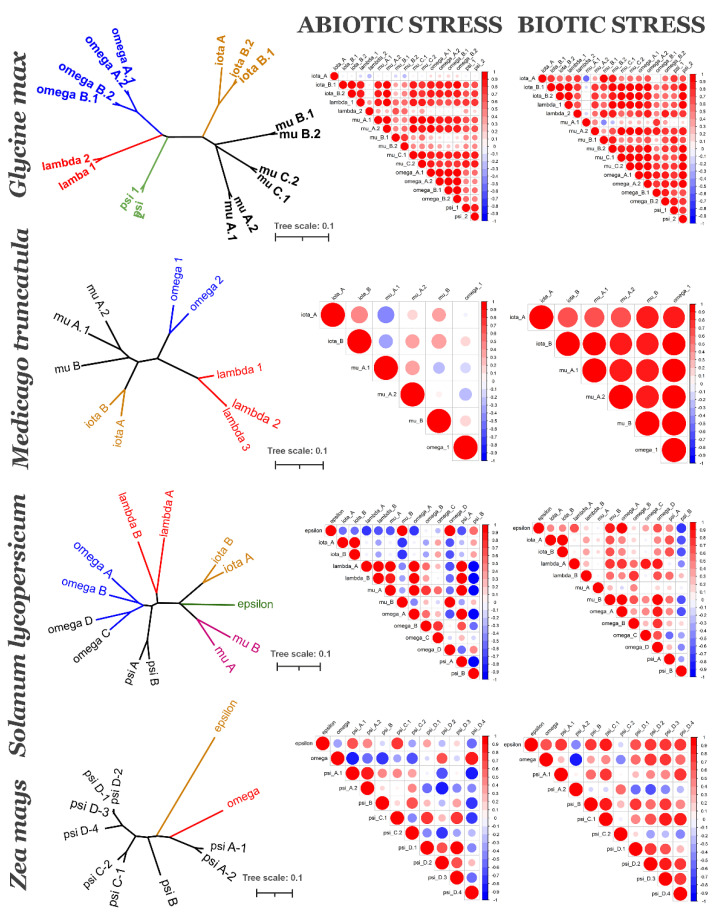
14-3-3 isoforms of four species: *Glycine max* (soybean), *Medicago truncatula* (barrel medic), *Solanum lycopersicum* (tomato) and *Zea mays* (maize): phylogenetic relationships (left graph) and coexpression under abiotic and biotic stress. Left graph is unrooted NJ tree of 14-3-3s, bar is number of nucleotide changes per site. Colour of a branch reflects different subfamilies groups. Correlation heatmaps show PCCs between each pair of 14-3-3s. Positive correlations are displayed in red, negative correlations are displayed in blue. The size of the circle and the colour intensity are proportional to the PCCs.

**Figure 5:**
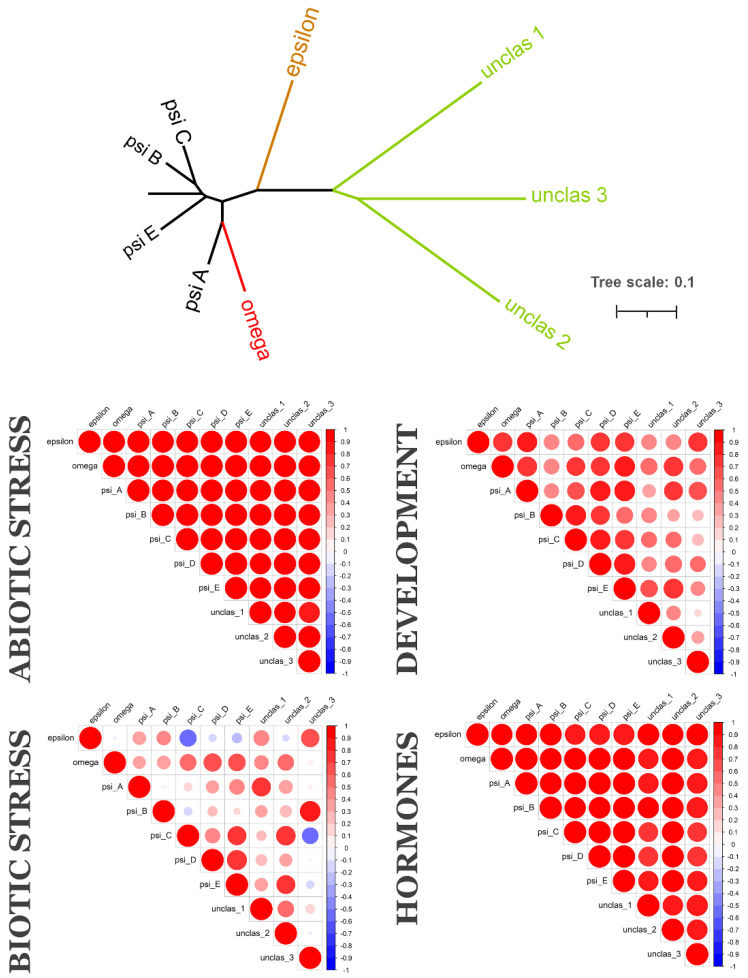
*Oryza sativa* 14-3-3 isoforms: phylogenetic relationships (upper graph) and coexpression under different physiological conditions (four correlation heatmaps). Upper graph is unrooted NJ tree of 14-3-3s from *O. sativa*, bar is number of nucleotide changes per site. Colour of a branch reflects different subfamilies groups. Correlation heatmaps show PCCs between each pair of 14-3-3s. Positive correlations are displayed in red, negative correlations are displayed in blue. The size of the circle and the colour intensity are proportional to the PCCs.

To understand if evolutionarily related 14-3-3 proteins are also functionally related, we analysed their coexpression. We employed two public databases for coexpression analyses. The PLANEX database provides genome-wide coexpression data, obtained via analyses of multiple microarrays, deposited in GEO NCBI [26]. The EXPath 2.0 database provides expression data from both microarray and RNAseq data [27]. PLANEX gives the Pearson correlation coefficient (PCC) for each pair of genes of interest, based on integrated analysis of all microarray experiments. EXPath 2.0 is a platform which can compare gene expression patterns in separated, different types of experiments. In the PLANEX we found PCCs for two monocots species, barley and rice, as well as three dicot species, soybean, tomato and *A. thaliana* (Figure S5). From EXPath 2.0, we retrieved expression data and calculated PCCs for *A. thaliana* (Figure 3), *Medicago truncatula*, soybean, tomato, maize (Figure 4) and rice (Figure 5).

*A. thaliana* is the most-studied plant species, and 14-3-3 proteins subfamilies’ classification is based on this species. *A. thaliana* has five epsilon-type isoforms and eight non-epsilons. Based on coexpression data (Figure 3), we can separate different functional groups of 14-3-3s in *A. thaliana*. Closely related kappa and lambda isoforms formed functional group with each other under abiotic stress, biotic stress, in different developmental stages, and during hormone actions. Another functional group, which was coexpressed under all studied physiological conditions, included epsilon isoform and three psi-group isoforms: nu, psi and upsilon. Under biotic stress and hormone actions, this group also included three omega-group isoforms. The mu isoform showed a negative correlation with most other isoforms under biotic stress and hormone actions, whereas in developmental studies iota, omicron and pi had negative correlations with other 14-3-3s. Integrated coexpression data, provided by PLANEX, partly confirmed the observed coexpression groups (Figure S5). First coexpression group included pi and iota, and showed a negative correlation with others isoforms, even with closely related (pi and epsilon—strong negative correlation, pi and omicron—no correlation). The second coexpression group included kappa/lambda from non-epsilon type and mu from epsilon. The third group included complete omega (omega, phi, chi) and psi (psi, upsilon, nu) groups of isoforms as well as the epsilon isoform itself. Omicron, which is another epsilon-type isoform, took a separate place and showed weak or absent correlation with other orthologs. Data from both databases confirm that the omega group, psi group and epsilon can form one functional group, and kappa and lamba another. Most of the epsilon-type isoforms showed negative correlations with non-epsilon and epsilon itself. Thus, closely related isoforms in *A. thaliana* seem to be involved in same physiological function. 

Let us turn now to other species. We retrieved expression data for two Fabaceae species: *Glycine max* (soybean) and *Medicago truncatula* (barrel medic). *G. max* had 17 isoforms: 9 of epsilon-type and 8 of non-epsilon type. There was not epsilon itself isoform in Fabaceae. The mu subfamily in *G. max* included six isoforms, which clustered into three different clades (Figure S1) and can be considered as orthologous: mu A, mu B, and mu C. Each group of orthologous included a paralogous pair of isoforms, denoted by numbers 1 and 2 (Figure 4). The iota subfamily includes two orthologous groups; one of each includes two paralogues. The omega subfamily includes two groups with two paralogues in each. Lambda and psi groups include each pair of paralogues. The majority of 14-3-3 isoforms in *G. max* showed high correlation in expression under both biotic and abiotic stress (Figure 4). There were only several exceptions. Under abiotic stress, isoform iota A showed slightly negative or slightly positive expression with others, mu B.1, mu B.2, and lambda 2 had slight correlation with others. Interestingly, mu B.1 and mu B.2 seem to be more ancient and closer to monocots than other mu isoforms. Mu A.1 showed negative or very low positive correlation of expression with other isoform under biotic stress. In PLANEX data (Figure S5), mu B.1 and mu B.2 showed no or weak negative correlation in expression with other isoforms, including other mu isoforms; strong correlations were found between other mu isoforms, psi, lambda and all omega isoforms. On PlANEX, iota isoforms formed their own functional group, whereas in EXPAth data, iota A had different expression level under abiotic stress condition.

Another Fabaceae species, *M. truncatula,* has only 11 isoforms of 14-3-3 proteins, and we found expression data only for 6 isoforms: iota A, iota B, mu A.1, mu A.2, mu B and omega 1 (Figure 4). There was neither information about any of three lambda isoforms, nor about omega 2 in the EXPath database. PLANEX did not contain data on *M. truncatula*. All observed isoforms showed high positive correlations under biotic stress conditions, and low positive or low negative (mu A.1) under abiotic stress. There is similarity between two Fabaceae species, both *G. max* and *M. truncatula* has mu A.1 negatively correlated with other isoforms, but under different conditions: biotic stress in *G. max* and abiotic stress in *M. truncatula* (Figure 4). 

The last Eudycotyledone species analysed was tomato *Solanum lycopersicum* from the Solanaceae family. *S. lycopersicum* has 13 isoforms of 14-3-3 proteins: 5 of epsilon type (1 epsilon itself, 2 iota, 2 mu) and 8 of non-epsilon type (2 lambda, 2 omega and 4 psi). Rather different functional groups formed together in terms of 14-3-3s under biotic and abiotic stress (Figure 4). Under abiotic stress conditions, the first functional group included both lambda isoforms, omega A, and psi A; the second group included epsilon, mu B, and omega D; the third included two iota isoforms. The second group showed negative correlations with almost all other isoforms. Under abiotic stress conditions, the first functional group included epsilon, mu B, omega A, omega D, and lambda A; the second group included both iota isoforms and psi A. Psi B showed a negative correlation with many others isoforms under both conditions. The negative correlation between expression of psi A and psi B is interesting, because it is a case of closely related isoforms showing an opposite expression pattern. In the PLANEX database we found data for nine 14-3-3 proteins of *S. lycopersicum* (Figure S5). It was possible to define two functional groups: (1) omega C and omega A; (2) omega D, epsilon, mu, lambda B, and psi A. There were no data on the psi B isoform. 

Turning to the monocotyledon species, *Zea mays* (Poaceae family) has 11 isoforms of 14-3-3 proteins. Like in other Poales, the main diversity of its 14-3-3s belongs to the psi group (9 of 11). Under abiotic stress conditions, there were four functional group: (1) epsilon and psi C.1; (2) omega, psi D.2, and psi D.4; (3) psi A.2 and psi A.1; (4) psi C.1, psi D.1, psi D2, and psi D.3. Omega, psi D.2, and psi D.4 showed negative correlation with many other isoforms. Under biotic stress conditions, expression of epsilon correlated with the most isoforms with the exception of psi A.2 and psi C.2. There were also other functional groups: psi B and psi C.1; all four psi D isoforms. 

*Oryza sativa,* like *Z. mays*, has only one epsilon and one omega isoform, but several psi isoforms (Figure 5). There were three sequences of 14-3-3-like proteins, which were too divergent, and we could not include them in Bayesian phylogeny, and therefore classified. These could be pseudogenized sequences, but we assessed data about their expression. It seems that all 14-3-3s in *O. sativa* had a similar expression response under abiotic stress and hormone action. In development studies, expressions of 14-3-3s also correlated in a positive manner. Under biotic stress conditions, the pattern of correlation expression was more diverse. Psi A correlated with unclassified 14-3-3 protein 1; psi C and Psi E with unclassified protein 2; psi B with unclassified protein 3; psi D with psi E. Negative correlation was demonstrated between epsilon and psi C, and psi C and unclassified protein 3 under biotic stress conditions. Microarray expression data analysed via PLANEX also showed strong correlation between 14-3-3 proteins in rice *O. sativa* (Figure S5). Almost no correlation or very weak negative correlation was found only between expression of one of the unclassified isoforms and other isoforms. 

We found coexpression data for six out of seven isoforms of *Hordeum vulgare* (barley) in the PLANEX database: one epsilon, one omega and four psi isoforms. We did not find strong correlation between expressions of six isoforms in barley (Figure S5). Medium correlation was observed between omega, and three psi isoforms. No or slightly negative correlations were found between the fourth psi isoform (psi C) and epsilon with all others isoforms.

To address more precisely the specific expression of 14-3-3 proteins, we employed a spatiotemporal database, CATchUP [28]. The information collected from this database is presented in Table 1. Unfortunately, it lacks the data about the majority of 14-3-3s. However, we can see that 14-3-3 from one subfamily is expressed in different species in different organs.

## 3. Discussion

In this study, we focused on the evolution of the family of 14-3-3 proteins in flower plants. We found that ancient as well as modern splits took place in the evolution of this gene family. A split between epsilon and non-epsilon isoforms seems to be the fundamental event in the evolution of 14-3-3 proteins in plants. Our phylogenetic analysis confirmed the ancient split between epsilon and non-epsilon types of isoforms. This split was detected in one of the first phylogenetic analyses, which included only 25 proteins from 9 plant species [29], and was confirmed later [14,18,21]. Exon–intron gene structure also correlates with division into epsilon and non-epsilon groups [30]. However, it was not clear when this split took place in evolutionary history, before division into eudicots and monocots [14], or later in a common ancestor of eudicots [21]. Sequences of epsilon-type 14-3-3 proteins were found in mosses and lycophyte *Selaginella* [18], suggesting that this split could happen even earlier, in a common ancestor of green plants.

Besides divergence between epsilon and non-epsilon, we found other ancient splits. Analysis of 14-3-3s in *Amborella* can point out which 14-3-3 subfamilies are the most ancient. *A. trichopoda* is a unique plant, the only species in the order Amborellales. It is a sister evolutionary branch to all other angiosperms and arose before the divergence monocots and eudicots [31]. *Amborella* has epsilon, iota, psi, and kappa 14-3-3-proteins (Figure S1, Table S3), thereafter, at least these four subfamilies were presented in the common ancestor of all angiosperm plants. Besides four well-known types of isoforms *Amborella* has six non-epsilon type isoforms, which formed a separate clade. These isoforms could be relicts of ancient non-epsilon type isoforms.

The diversity of isoforms of 14-3-3 proteins in plants is a result of gene duplication events. There were at least 106 whole-genome duplications (WGDs) in angiosperm evolution [32], and a lot of small-scale genome duplications (SSDs) in various plant lineages [33]. The early duplication event occurred in a common ancestor of green plants and lead to formation of pro-epsilon and pro-non-epsilon isoforms. Pro-epsilon probably was close to modern iota isoforms, whereas pro-non-epsilon is more similar with modern kappa isoforms. More late duplication events lead to the formation of iota and epsilon isoforms from pro-epsilon, and kappa and psi from pro-non-epsilon. The main WGDs events in plant history are denoted by Greek letters, and so as not to confuse them with 14-3-3 subfamilies, we use Greek symbols for WGDs and English-transliterated letters for 14-3-3s. The formation of iota, epsilon, kappa and psi subfamilies can be connected with the following WGDs: the ζ event that happened in a in common ancestor of seeds plants 320-350 million years ago or the ε event that happened in a common ancestor of angiosperm plants 190–235 million years ago [34,35].

We did not find mu and omega isoforms in *Amborella*, but there were mu and omega isoforms in monocots. Therefore, formation of mu and omega subfamilies could arise after separation of the common ancestor of monocots and eudicots from basal angiosperms through SSDs, while WSDs events have been not yet identified in the period between *Amborella* separation and monocotyledons–eudicotyledons diversification [30,32].

Ancient duplication followed by recent duplications has resulted in a high diversity of isoforms within subfamilies. The origin of epsilon, pi, omicron, upsilon, nu, psi, chi, phi, omega, kappa and lambda 14-3-3 proteins in *Arabidopsis,* explained by two recent α/β WGDs, took place in common ancestor of Brassicaceae family circa 14 and 80 million years ago [32,34,35]. *Carica papaya*, which is also part of Brassicales order, but from another family, has only one isoform in the epsilon and omega group.

At least three duplications produced high diversity of psi-group isoforms in Poaceae: the ρ, σ, and τ events [34]. The τ event is a WGD that happened 110–135 million years ago in the common ancestor of the biggest part of the monocot plants, including grasses, orchids, palms, bananas, bamboo, and pineapple [34]. The σ event took place in the common ancestor of Poales order, which includes grasses, sedges, bromeliads, etc.; whereas the ρ event happened later in an ancestor of the grass family Poaceae itself [36]. The vast variety of 14-3-3s in banana (25 isoforms) [17] could be driven by a combination of the τ event and three independent lineage-specific WGDs which happened 65–100 million years ago after the separation of banana’s ancestor from Poales [37]. The formation of new gene pairs due to lineage WGDs was shown in banana for several gene families: 9-*cis*-epoxycarotenoid dioxygenase and sucrose-phosphate synthase [38].

Most of the 14-3-3 family gene duplications could be explained by WGD events, which happened many times during repeated cycles of “hybridization-polyploidization-secondary diploidization”, typical for plant evolution [39]. However, the variety of plant 14-3-3 proteins observed in angiosperms plants is explained not only by duplication, but also by lineage-specific gene loss. For example, the Poaceae family (grasses) lost mu and kappa isoforms, and all Poales (grasses and pineapple) lost the iota subfamily, and the Fabaceae family lost the epsilon-group isoforms. The loss of whole subfamilies of important proteins in plant lineages is not unique for 14-3-3 proteins. For example, monocots lost two subfamilies of transcription factor 3R-MIYR [40]. During the cycles of “hybridization-polyploidization-secondary diploidization”, polyploidization is often followed by loss of genetic materials, from individual genes to whole chromosomes [39].

14-3-3 proteins play a fundamental role in the regulation of plant physiology, thus there is a question of how species cope with a loss of whole 14-3-3 subfamilies. It is still unknown if 14-3-3 proteins are involved in physiological function in an isoform-specific manner. In an interactome study on developing *A. thaliana*’s seeds, 27 non-specific proteins were found to interact with 14-3-3s, 28 chi-specific (non-epsilon group) and 49 epsilon-specific [41]. Non-epsilon isoforms omega, kappa, lambda and chi better bind with plasma membrane H^+^-ATPase than epsilon, mu and omicron isoforms [42]. Different isoforms of 14-3-3 proteins show variation in binding activity with the C-terminus of plasma membrane H^+^-ATPase. According to the Plant Interactome Database [43,44] and PlaPPISite [45,46,47] the plasma membrane H^+^-ATPases of *A. thaliana* are interacting with six 14-3-3 proteins. The maximal sites of interaction were shown for AHA2 and much less for AHA6 and AHA10. Evolutionarily close phi and omega isoforms demonstrate an opposite capability for binding H^+^-ATPase [14]. The epsilon isoform in *A. thaliana* works as a positive regulator in cold-stress adaptation, lambda works as negative [48,49], but the epsilon-like isoform in rice is not affected by low-temperature stress [50].

Most studies on functional specificity of 14-3-3 isoforms in plants have been carried out on *A. thaliana*. There is some similarity in expression profiles of 14-3-3 in different plant species, for example iota isoforms are highly expressed in floral organs of both *Arabidopsis* and poplar [22]. However, the current study found that 7 out of 13 isoforms are specific for *Arabidopsis* and the Brassicaceae family. Subsequently, it was not possible to generalize all data available for *A. thaliana* to other plant species. Coexpression data (Figure 3, Figure 4 and Figure 5) shows that each species has its own coexpression pattern for 14-3-3 proteins. The more divergent taxonomically they are, the more different functional groups formed 14-3-3 proteins. In *A. thaliana,* closely related isoforms from omega, psi and kappa groups had similar coexpression patterns under almost all physiological conditions. On the other hand, pi and omicron, which are closely related to epsilon and included in the epsilon group, showed negative correlation in expression with epsilon. In other species, closely related isoforms were often coexpressed, but not always. For example, two psi isoforms in tomato showed contrary expression patterns (Figure 4).

It seems that the epsilon isoform is very important, for instance, in *A. thaliana* it involved in the stress reaction, hormone action and development. In some other species (*Z. mays*, *S. lycopersicum*), epsilon isoforms are also involved in functional groups connected with abiotic and biotic stress responses. It is interesting that Fabaceae lost epsilon isoforms during their evolution; other isoforms should thus replace their functions. Another important fact is that that Fabaceae species have symbiotic relationships with nitrogen-fixing bacteria, and during interaction form a special root organ—a nodule [51]. Therefore, some 14-3-3s should be involved in regulation of physiological processes that take place in nodules. In soybean, the isoform mu A.1 is specifically expressed in nodules but also in the plant embryo (Table 1). A possible explanation for this might be that the protein that existing in a species, has begun to execute a new function. After WGDs or SSDs, many duplicated genes obtained novel functions through gain-of-function mutations in both coding and regulatory regions [52]. For example, *TEL* gene family was lost in several plant lineages, but in *Arabidopsis* these genes duplicated and gained new functions [53]. We suggested that lineage-specific gene loss is compensated by a functional plasticity. Duplicated isoforms from other subfamilies probably take the function of lost isoforms or take a new function, such as in the case of Fabaceae endosymbiosis.

## 4. Materials and Methods

### 4.1. 14-3-3 Sequences Search

The majority of sequences of plant 14-3-3 proteins were retrieved from the Phytozome 12 database (https://phytozome.jgi.doe.gov/ (accessed on 13 July 2021) The search was performed in the 46 plant genomes released by DOE-JGI (Table S1) using “14-3-3” as a keyword. 14-3-3 proteins of *Nicotiana tabacum* and *Saccharomyces cerevisiae* were found in UniProt database [54], and corresponding nucleotide sequences were retrieved from GenBank NCBI (www.ncbi.nlm.nih.gov/genbank/ (accessed on 31 October 2021). Obtained sequences were checked for presence of conservative 14-3-3 PFAM domain PF00244. We excluded sequences which had other functional domains, and sequences with nontypical-for-14-3-3 protein length (219–349 amino acids). A list of analysed sequences is presented in the Supplementary Table S2.

### 4.2. Phylogenetic Analysis

For phylogenetic analysis we used protein-coding nucleotide sequences, because the phylogenies based on amino acid sequences have many clades with poor statistical support [14,20]. An increase in the number of characters in an input data matrix improves tree topology in the case of 14-3-3 proteins (for example [18]). Sequences were aligned by MAFFT 7.2 using L-INS-i strategy [55]. Hypervariable 5′ and 3′ ends (matching N- and C-termini in amino acid sequences) were trimmed. Trimmed regions were so diverse that it was not possible to find homologic nucleotides and make proper alignment [29]. The sequence of yeast 14-3-3 protein BMH1 (UniProt A6ZRD4) was used as an outgroup in phylogenetic analysis. For manual inspection of the alignment, filtering and trimming, we used AliView software [56].

We employed two contrasting tree-building approaches. A neighbour-joining tree was constructed using MEGA X [57] with 1000 bootstrap replicates. A Bayesian tree was constructed with MrBayes v. 3.2.7 [58] using the General Time Reverse model with Gamma. The best nucleotide substitution model was chosen using the Bayesian information criterion (BIC), as implemented in the MEGA X. We performed Bayesian analysis with two runs, each with four chains of 2.5 million MCMC generations, sampling every 100 generations, and with 25% burn-in. Posterior probability values were used as statistical support for branches. For graphical presentations of phylogenetic trees, iTOL web tool was used [59]. Phylogenetic analysis was repeated several times.

### 4.3. Coexpression and Spatiotemporal Expression Analysis

To analyse coexpression of different 14-3-3 isoforms, we employed databases PLANEX [26] and ExPath 2.0 [27]. From the PLANEX database we retrieved Pearson’s correlation coefficients (PCCs) for coexpression of 14-3-3s in rice *Oryza sativa*, soybean *Glycine max*, barley *Hordeum vulgare*, tomato *Solanum lycopersicum* and *Arabidopsis thaliana* using Gene IDs and nucleotide Blast search. We accessed this database on the webpage http://planex.plantbioinformatics.org (last accessed on 22 June 2021, current version of PLANEX is being moved to http://planex.plantgenomicslab.org/, accessed on 29 November 2021). From the ExPath 2.0 database in the expression comparison mode we exported expression data for 14-3-3s of *Arabidopsis thaliana, Oryza sativa*, *Zea mays*, *Glycine max*, *Medicago truncatla*, and *Solanum lycopersicum.* We exported relative expression data of RNAseq experiments, and normalized expression data from microarrays. We calculated PCCs, which then we visualized using corrplot package for R [60,61]. For spatiotemporal expression comparison we employed the CATchUP database [28]. In this database, we found data for several 14-3-3s of *Arabidopsis thaliana, Glycine max*, *Medicago truncatla*, *Solanum lycopersicum,* and *Sorghum bicolor.*

## 5. Conclusions

The 14-3-3 proteins are highly conserved proteins, widespread in main groups of eukaryotes. Regardless of their conservativeness and universalism, 14-3-3s are involved in different physiological functions in plants and animals. In plants opposed to animals and fungi, the 14-3-3 family includes a high number of isoforms. Phylogenetic analysis together with coexpression analysis revealed that 14-3-3s evolved in plants mainly via duplication, followed by lineage-specific gene loss and neofunctionalization. Formation of iota, mu, epsilon, omega, psi and kappa subfamilies of 14-3-3s is connected with ancient WGDs, which occurred in common ancestors of seed plants and angiosperm plants. Formation of isoform diversity within subfamilies can be explained by lineage-specific WGDs and SSDs. Evolution of the 14-3-3 family is closely related with recurrent cycles of polyploidization, which are one of the most important driving forces in angiosperm evolution. It is possible to hypothesise that lineage-specific gene loss (such as loss of epsilon in Fabaceae and loss of mu and iota in Poaceae) could be compensated by defunctionalisation of remaining isoforms. Coexpression patterns suggested that each species obtained their own functional groups of 14-3-3 proteins. To develop a full picture of 14-3-3 protein regulatory functions, it is important not only to extrapolate model plant data, but to investigate 14-3-3s in various species.

## Figures and Tables

**Table 1:** Spatiotemporal data on specific expression of 14-3-3s according to CATchUP database.

Subfamily of 14-3-3 Protein	Species	Gene ID	Classification	Specific Expression Location and Conditions
Omega group	*A. thaliana*	AT4G09000	Chi (GRF (1)	Nonhair root epidermal cell
	*G. max*	Glyma.14G176900	Omega B.2	Shoot system; drought environment
Psi group	*S. bicolor*	Sobic.005G145200	Psi	Anther
Kappa/lambda group	*A.thaliana*	AT5G10450	Lambda (GRF 6)	Whole plant
	*G. max*	Glyma.04G092600	Lambda 1	Mature leaves in full sunlight
	*M. truncatula*	Medtr3g100620.1	Lambda 3	Flower, lamina
Epsilon group	*S. bicolor*	Sobic.005G183200	Epsilon	Root; abscisic acid treatment
Iota Group	*S. lycopersicon*	Solyc05g012420	Iota B	Flower bud
	*G. max*	Glyma.20G025900	Iota A	Flower
	*G. max*	Glyma.02G115900	Iota B.2	Mature leaves in full sunlight
Mu group	*G. max*	Glyma.05G158100	Mu A.1	Root noduleplant embryo axis
	*G. max*	Glyma.12G229200	Mu B.1	Inner integument
	*G. max*	Glyma.13G270600	Mu B.2	Seed coat

## Data Availability

Not applicable.

## Supplementary Materials

The following are available online at https://www.mdpi.com/article/10.3390/plants10122724/s1, Figure S1: Phylogenetic tree (cladogram) of plant 14-3-3 proteins family based on Bayesian analysis of coding nucleotide sequences. Branches are labelled with Bayesian support values. Section colours indicate subfamilies, Table S1: List of the source genomes used for 14-3-3 search, Table S2: Sequences used for the analyses; Table S3: Arabidopsis-based classification of analysed 14-3-3 isoforms. Dataset S4: Multiple alignment of conservative region of coding part of plant 14-3-3 genes. Figure S5 Coexpression of 14-3-3s in *A. thaliana*, *S. lycopersicon*, *G. max*, *H. vulgare* and *O. sativa* inferred from PLANEX database.
