# Supplementary material for: Evolution of 14-3-3 Proteins in Angiosperm Plants: Recurring Gene Duplication and Loss
Source: Plants (Basel). 2021 Dec 11;10(12):2724. doi: 10.3390/plants10122724 (PMC8703263; doi:10.3390/plants10122724)
Supplement: Supplementary file 1 [file plants-10-02724-s001.zip › Figure S5.pdf]

Figure S5
Co-expression of 14-3-3s in *A. thaliana*, *S. lycopersicon*, *G. max*, *H. vulgare* and *O. sativa* inferred from PLANEX database.

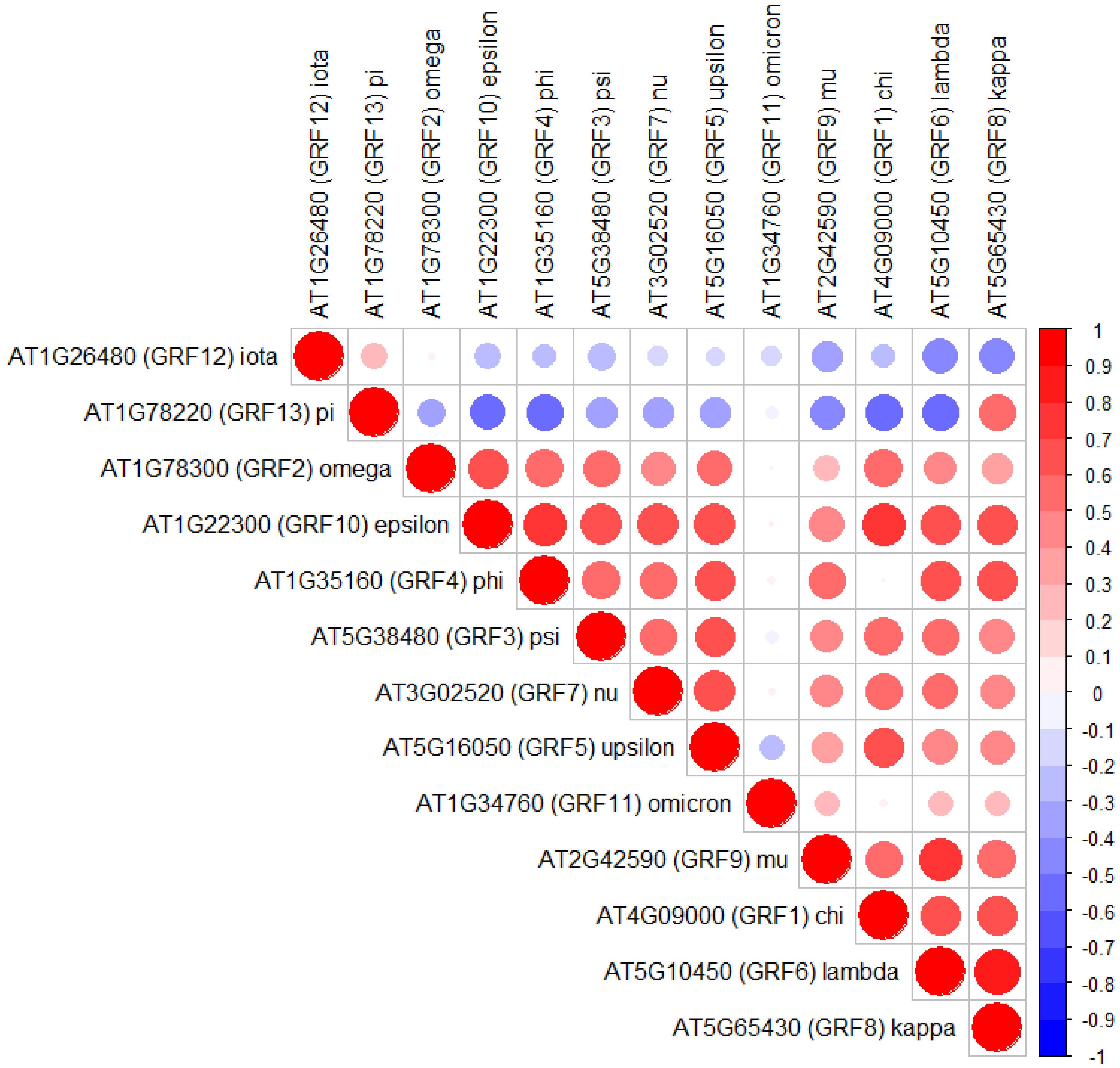


Co-expression of 14-3-3 proteins in *Arabidopsis thaliana*. Correlation heatmaps show PCCs between each pair of 14-3-3s. Positive correlations are displayed in red colour, negative correlations are displayed in blue colour. The size of the circle and the colour intensity are proportional to the PCCs.

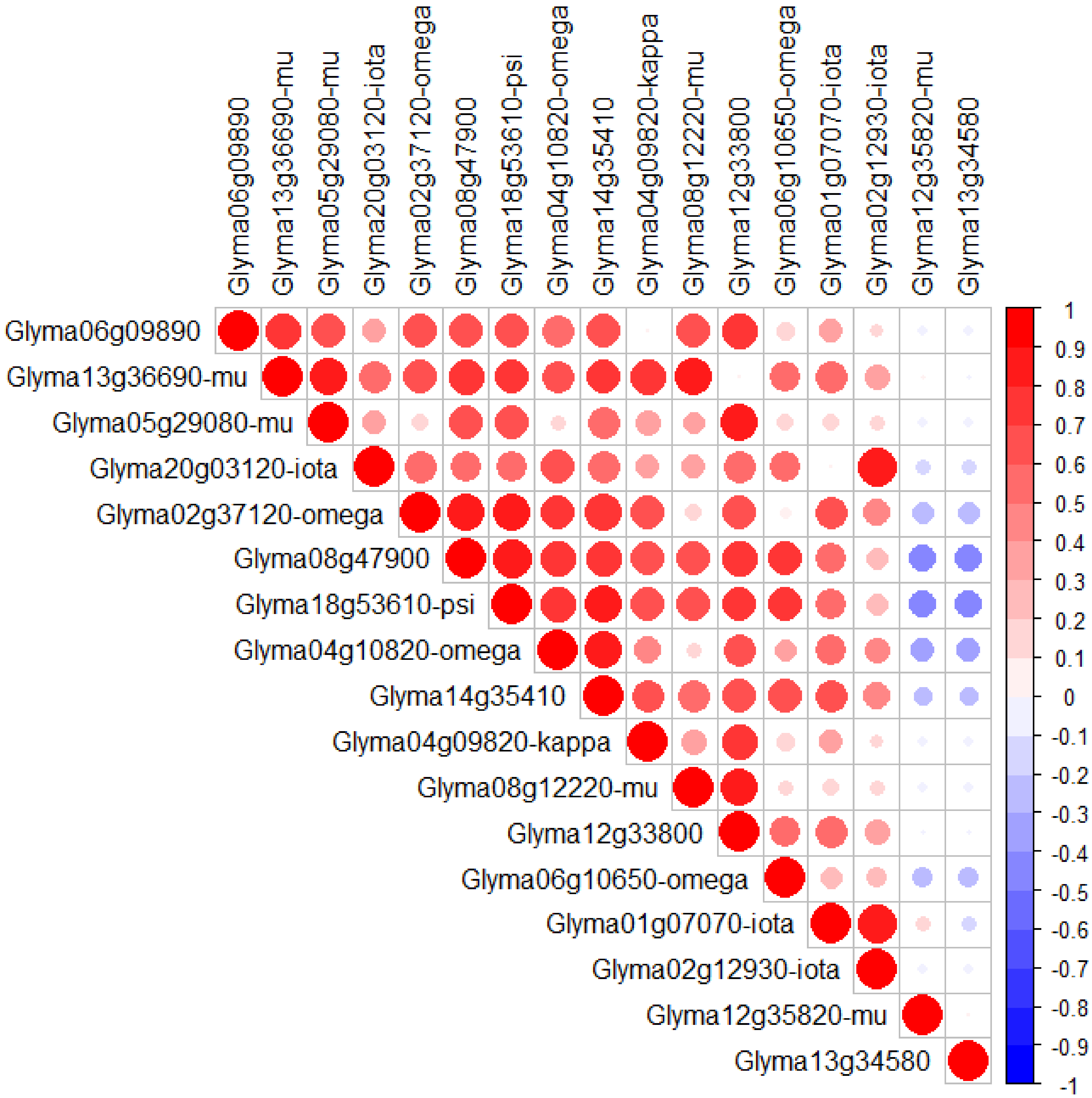


Co-expression of 14-3-3 proteins in *Glycine max*. Correlation heatmaps show PCCs between each pair of 14-3-3s. Positive correlations are displayed in red colour, negative correlations are displayed in blue colour. The size of the circle and the colour intensity are proportional to the PCCs.

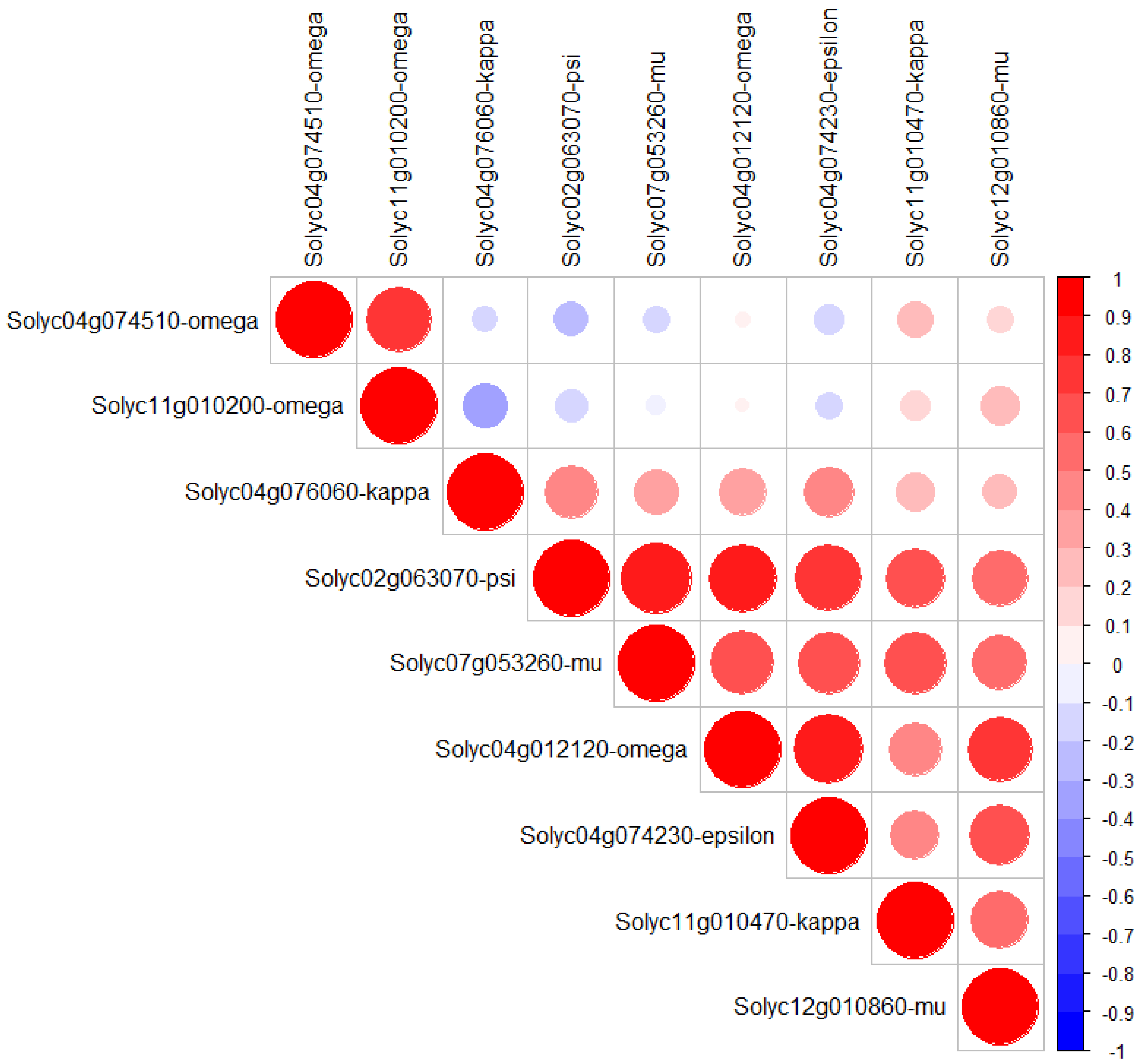


Co-expression of 14-3-3 proteins in *Solanum lycopersicum*. Correlation heatmaps show PCCs between each pair of 14-3-3s. Positive correlations are displayed in red colour, negative correlations are displayed in blue colour. The size of the circle and the colour intensity are proportional to the PCCs.

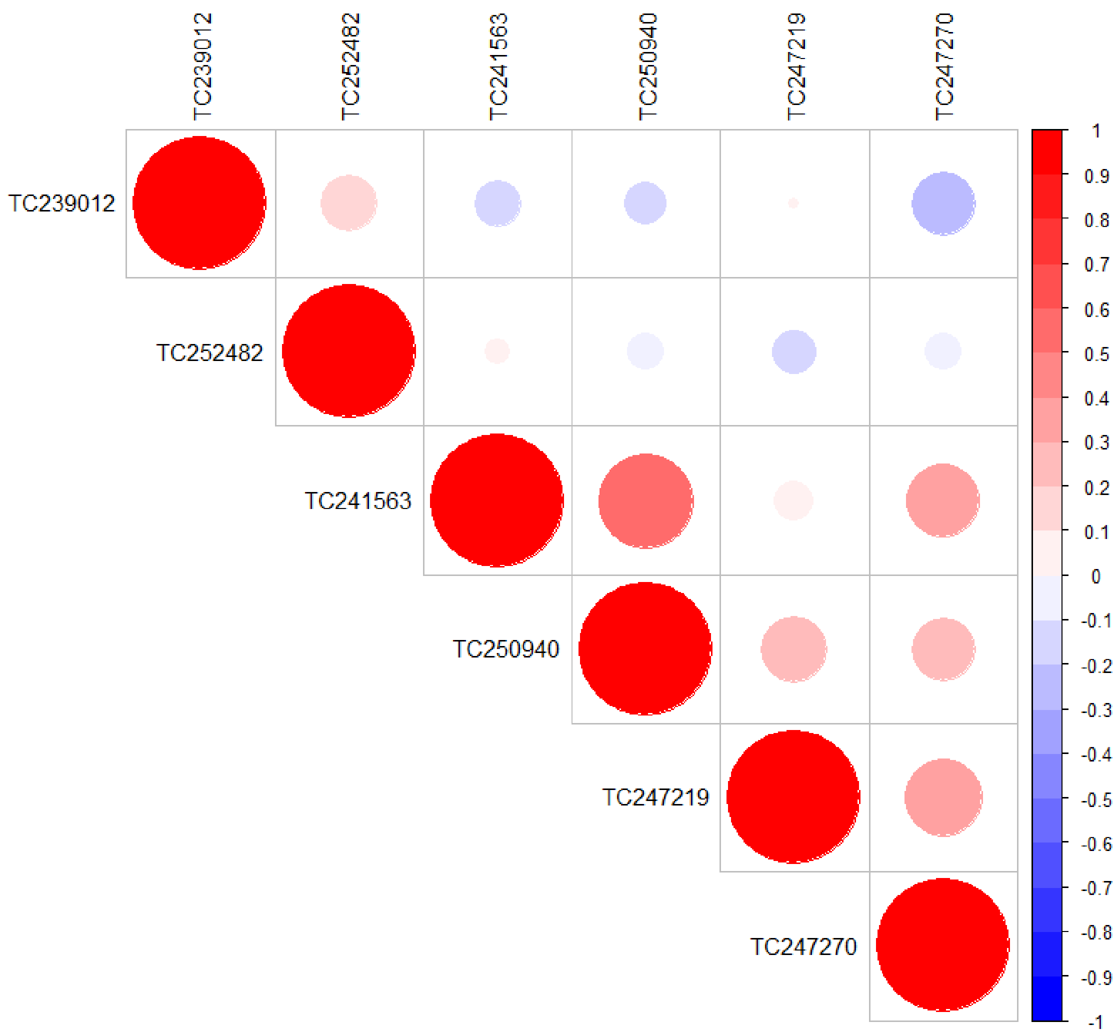


Co-expression of 14-3-3 proteins in *Hordeum vulgare*. Correlation heatmaps show PCCs between each pair of 14-3-3s. Positive correlations are displayed in red colour, negative correlations are displayed in blue colour. The size of the circle and the colour intensity are proportional to the PCCs. Subfamily classification: TC250940 – omega, TC252482 – epsilon, TC241563 – psi A, TC247219 – Psi B, TC239012 – psi C, TC247270 – Psi E

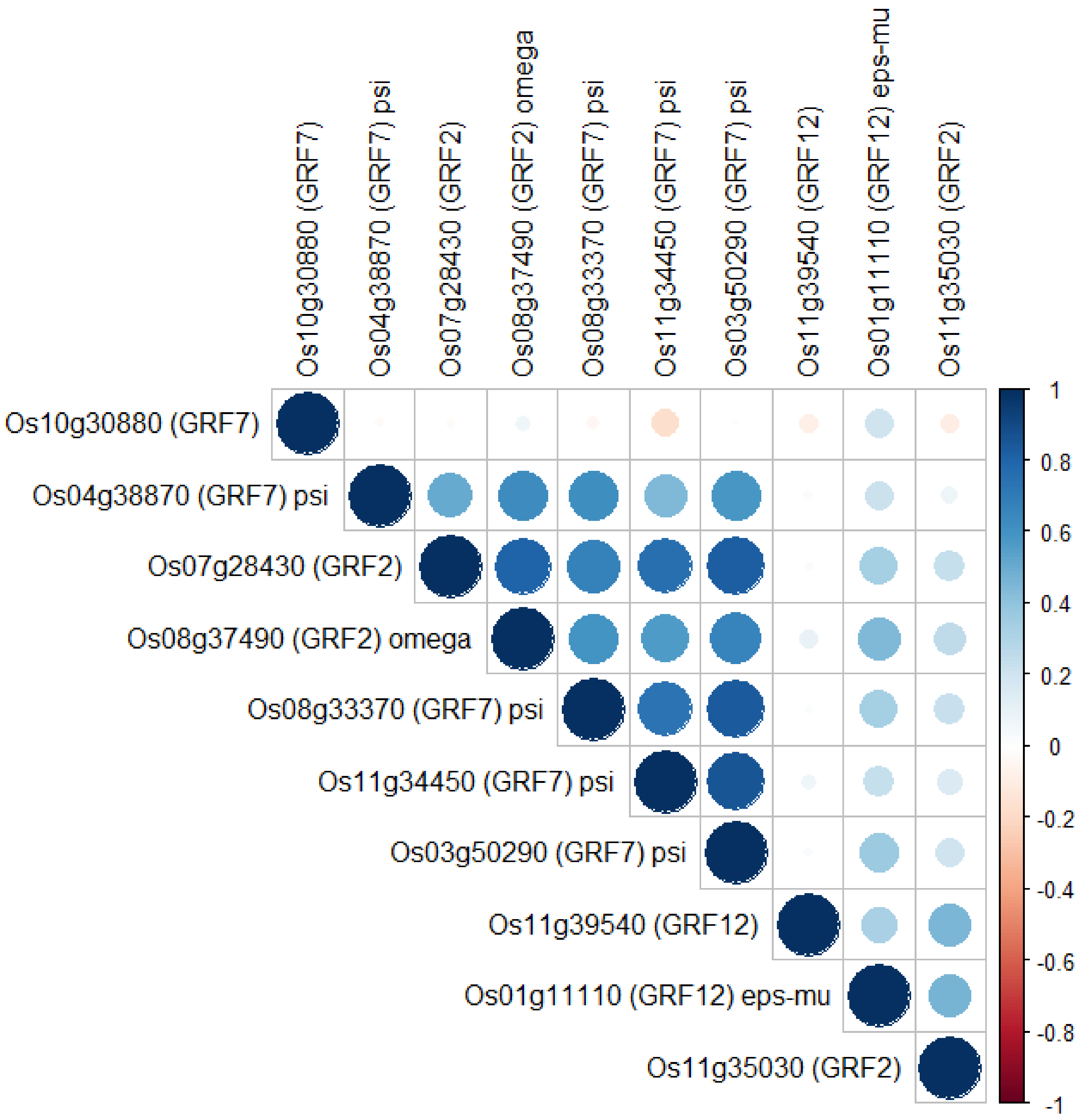


Co-expression of 14-3-3 proteins in *Oryza sativa*. Correlation heatmaps show PCCs between each pair of 14-3-3s. Positive correlations are displayed in blue colour, negative correlations are displayed in red colour. The size of the circle and the colour intensity are proportional to the PCCs.
