# Supplementary material for: Evolution of 14-3-3 Proteins in Angiosperm Plants: Recurring Gene Duplication and Loss
Source: Plants (Basel). 2021 Dec 11;10(12):2724. doi: 10.3390/plants10122724 (PMC8703263; doi:10.3390/plants10122724)
Supplement: Supplementary file 1 [file plants-10-02724-s001.zip › Table S1.pdf]

Table S1. Source genomes used for 14-3-3 search.

Phytozome genome release	Reference
1. *Amaranthus hypochondriacus* v1.0	[1]
2. *Amborella trichopoda* v1.0	[2]
3. *Ananas comosus* v3	[3]
4. *Aquilegia coerulea* v3.1	[4]
5. *Arabidopsis halleri* v 1.1	Arabidopsis halleri v1.1, DOE-JGI, http://phytozome.jgi.doe.gov/
6. *Arabidopsis lyrata* v2.1	[5]
7. *Arabidopsis thaliana* TAIR10	[6]
8. *Boechera stricta* v1.2	Boechera stricta v1.2, DOE-JGI, http://phytozome.jgi.doe.gov
9. *Brachypodium distachyon* Bd21-3 v1.1	Brachypodium distachyon Bd21-3 v1.1 DOE-JGI, http://phytozome.jgi.doe.gov/
10. *Brachypodium stacei* v1.1	Brachypodium stacei v1.1 DOE-JGI, http://phytozome.jgi.doe.gov/
11. *Brassica oleracea capitata* v1.0	[7]
12. *Brassica rapa* FPsc v1.3	Brassica rapa FPsc v1.3, DOE-JGI, http://phytozome.jgi.doe.gov/
13. *Capsella grandiflora* v1.1	[8]
14. *Capsella rubella* v1.0	[8]
15. *Carica papaya* ASGPBv0.4	[9]
16. *Citrus clementina* v1.0	[10]
17. *Citrus sinensis* v1.1	[10]
18. *Cucumis sativus* v1.0	https://phytozome.jgi.doe.gov
19. *Daucus carota* v2.0	[11]
20. *Eucalyptus grandis* v2.0	[12]
21. *Eutrema salsugineum* v1.0	[13]
22. *Fragaria vesca* v1.1	[14]
23. *Glycine max* Wm82.a2.v1	[15]
24. *Gossypium raimondii* v2.1	[16]
25. *Hordeum vulgare r1*	[17]
26. *Linum usitatissimum* v1.0	[18]
27. *Malus domestica* v1.0	[19]
28. *Manihot esculenta* v6.1	[20]
29. *Medicago truncatula* Mt4.0v1	[21]
30. *Mimulus guttatus* v2.0	[22]
31. *Musa acuminata* v1	[23]
32. *Oryza sativa* v7_JGI	[24]
33. *Panicum hallii* v2.0	[25]
34. *Panicum virgatum* v1.1	Panicum virgatum v1.1, DOE-JGI, http://phytozome.jgi.doe.gov/
35. *Phaseolus vulgaris* v2.1	Phaseolus vulgaris v2.1, DOE-JGI and USDA-NIFA, http://phytozome.jgi.doe.gov/
36. *Populus trichocarpa* v3.0	https://phytozome.jgi.doe.gov
37. *Prunus persica* v2.1	[26]

38. Ricinus communis v0.1	[27]
39. Salix purpurea v1.0	[28]
40. Setaria italica v2.2	[29]
41. Setaria viridis v1.1	[30]
42. *Solanum lycopersicum* ITAG4.0	[31]
43. Solanum tuberosum v4.03	[32]
44. Sorghum bicolor v3.1.1	[33]
45. Theobroma cacao v1.1	[34]
46. Trifolium pratense v2	[35]
47. Triticum aestivum v 2.2	[36]
48. Zea mays Ensembl-18	[37]

Reference

1. Clouse, J.W.; Adhikary, D.; Page, J.T.; Ramaraj, T.; Deyholos, M.K.; Udall, J.A.; Fairbanks, D.J.; Jellen, E.N.; Maughan, P.J. The Amaranth Genome: Genome, Transcriptome, and Physical Map Assembly. *Plant Genome* **2016**, *9*, plantgenome2015.07.0062, doi:10.3835/PLANTGENOME2015.07.0062.

2. DePamphilis, C.W.; Palmer, J.D.; Rounsley, S.; Sankoff, D.; Schuster, S.C.; Ammiraju, J.S.S.; Barbazuk, W.B.; Chamala, S.; Chanderbali, A.S.; Determann, R.; et al. The Amborella Genome and the Evolution of Flowering Plants. *Science* **2013**, *342*, doi:10.1126/science.1241089.

3. Ming, R.; VanBuren, R.; Wai, C.M.; Tang, H.; Schatz, M.C.; Bowers, J.E.; Lyons, E.; Wang, M.-L.; Chen, J.; Biggers, E.; et al. The Pineapple Genome and the Evolution of CAM Photosynthesis. *Nat. Genet.* **2015**, *47*, 1435, doi:10.1038/NG.3435.

4. Filiault, D.L.; Ballerini, E.S.; Mandáková, T.; Aköz, G.; Derieg, N.J.; Schmutz, J.; Jenkins, J.; Grimwood, J.; Shu, S.; Hayes, R.D.; et al. The Aquilegia Genome Provides Insight into Adaptive Radiation and Reveals an Extraordinarily Polymorphic Chromosome with a Unique History. *Elife* **2018**, *7*, doi:10.7554/ELIFE.36426.

5. Rawat, V.; Abdelsamad, A.; Pietzenuk, B.; Seymour, D.K.; Koenig, D.; Weigel, D.; Pecinka, A.; Schneeberger, K. Improving the Annotation of Arabidopsis Lyrata Using RNA-Seq Data. *PLoS One* **2015**, *10*, doi:10.1371/JOURNAL.PONE.0137391.

6. Lamesch, P.; Berardini, T.Z.; Li, D.; Swarbreck, D.; Wilks, C.; Sasidharan, R.; Muller, R.; Dreher, K.; Alexander, D.L.; Garcia-Hernandez, M.; et al. The Arabidopsis Information Resource (TAIR): Improved Gene Annotation and New Tools. *Nucleic Acids Res.* **2012**, *40*, D1202, doi:10.1093/NAR/GKR1090.

7. Liu, S.; Liu, Y.; Yang, X.; Tong, C.; Edwards, D.; Parkin, I.A.P.; Zhao, M.; Ma, J.; Yu, J.; Huang, S.; et al. The Brassica Oleracea Genome Reveals the Asymmetrical Evolution of Polyploid. *Nat. Commun.* **2014**, *5*, doi:10.1038/NCOMMS4930.

8. Slotte, T.; Hazzouri, K.M.; Ågren, J.A.; Koenig, D.; Maumus, F.; Guo, Y.-L.; Steige, K.; Platts, A.E.; Escobar, J.S.; Newman, L.K.; et al. The Capsella Rubella Genome and the Genomic Consequences of Rapid Mating System Evolution. *Nat. Genet. 2013 457* **2013**, *45*, 831–835, doi:10.1038/ng.2669.

9. Ming, R.; Hou, S.; Feng, Y.; Yu, Q.; Dionne-Laporte, A.; Saw, J.H.; Senin, P.; Wang, W.; Ly, B. V.; Lewis, K.L.T.; et al. The Draft Genome of the Transgenic Tropical

Fruit Tree Papaya (Carica Papaya Linnaeus). *Nature* **2008**, *452*, 991, doi:10.1038/NATURE06856.

10. Wu, G.A.; Prochnik, S.; Jenkins, J.; Salse, J.; Hellsten, U.; Murat, F.; Perrier, X.; Ruiz, M.; Scalabrin, S.; Terol, J.; et al. Sequencing of Diverse Mandarin, Pummelo and Orange Genomes Reveals Complex History of Admixture during Citrus Domestication. *Nat. Biotechnol.* **2014**, *32*, 656, doi:10.1038/NBT.2906.
11. Iorizzo, M.; Ellison, S.; Senalik, D.; Zeng, P.; Satapoomin, P.; Huang, J.; Bowman, M.; Iovene, M.; Sanseverino, W.; Cavagnaro, P.; et al. A High-Quality Carrot Genome Assembly Provides New Insights into Carotenoid Accumulation and Asterid Genome Evolution. *Nat. Genet. 2016 486* **2016**, *48*, 657–666, doi:10.1038/ng.3565.
12. Myburg, A.A.; Grattapaglia, D.; Tuskan, G.A.; Hellsten, U.; Hayes, R.D.; Grimwood, J.; Jenkins, J.; Lindquist, E.; Tice, H.; Bauer, D.; et al. The Genome of Eucalyptus Grandis. *Nat. 2014 5107505* **2014**, *510*, 356–362, doi:10.1038/nature13308.
13. Yang, R.; David, J.; Hao, C.; Mark, B.; Jane, G.; Jerry, J.; ShengQiang, S.; Simon, P.; Mingming, X.; Chuang, M.; et al. The Reference Genome of the Halophytic Plant Eutrema Salsugineum. *Front. Plant Sci.* **2013**, *4*, 46, doi:10.3389/FPLS.2013.00046.
14. Shulaev, V.; Sargent, D.J.; Crowhurst, R.N.; Mockler, T.C.; Folkerts, O.; Delcher, A.L.; Jaiswal, P.; Mockaitis, K.; Liston, A.; Mane, S.P.; et al. The Genome of Woodland Strawberry (Fragaria Vesca). *Nat. Genet.* **2011**, *43*, 109, doi:10.1038/NG.740.
15. Schmutz, J.; Cannon, S.B.; Schlueter, J.; Ma, J.; Mitros, T.; Nelson, W.; Hyten, D.L.; Song, Q.; Thelen, J.J.; Cheng, J.; et al. Genome Sequence of the Palaeopolyploid Soybean. *Nat. 2010 4637278* **2010**, *463*, 178–183, doi:10.1038/nature08670.
16. Paterson, A.H.; Wendel, J.F.; Gundlach, H.; Guo, H.; Jenkins, J.; Jin, D.; Llewellyn, D.; Showmaker, K.C.; Shu, S.; Udall, J.; et al. Repeated Polyploidization of Gossypium Genomes and the Evolution of Spinnable Cotton Fibres. *Nat. 2012 4927429* **2012**, *492*, 423–427, doi:10.1038/nature11798.
17. Beier, S.; Himmelbach, A.; Colmsee, C.; Zhang, X.-Q.; Barrero, R.A.; Zhang, Q.; Li, L.; Bayer, M.; Bolser, D.; Taudien, S.; et al. Construction of a Map-Based Reference Genome Sequence for Barley, Hordeum Vulgare L. *Sci. Data* **2017**, *4*, doi:10.1038/SDATA.2017.44.
18. Wang, Z.; Hobson, N.; Galindo, L.; Zhu, S.; Shi, D.; McDill, J.; Yang, L.; Hawkins, S.; Neutelings, G.; Datla, R.; et al. The Genome of Flax (Linum Usitatissimum) Assembled de Novo from Short Shotgun Sequence Reads. *Plant J.* **2012**, *72*, 461–473, doi:10.1111/J.1365-313X.2012.05093.X.
19. Velasco, R.; Zharkikh, A.; Affourtit, J.; Dhingra, A.; Cestaro, A.; Kalyanaraman, A.; Fontana, P.; Bhatnagar, S.K.; Troggio, M.; Pruss, D.; et al. The Genome of the Domesticated Apple (Malus × Domestica Borkh.). *Nat. Genet. 2010 4210* **2010**, *42*, 833–839, doi:10.1038/ng.654.
20. Bredeson, J. V; Lyons, J.B.; Prochnik, S.E.; Wu, G.A.; Ha, C.M.; Edsinger-Gonzales, E.; Grimwood, J.; Schmutz, J.; Rabbi, I.Y.; Egesi, C.; et al. Sequencing Wild and Cultivated Cassava and Related Species Reveals Extensive Interspecific Hybridization and Genetic Diversity. *Nat. Biotechnol. 2016 345* **2016**, *34*, 562–570, doi:10.1038/nbt.3535.

21. Young, N.D.; Debellé, F.; Oldroyd, G.E.D.; Geurts, R.; Cannon, S.B.; Udvardi, M.K.; Benedito, V.A.; Mayer, K.F.X.; Gouzy, J.; Schoof, H.; et al. The Medicago Genome Provides Insight into the Evolution of Rhizobial Symbioses. *Nature* **2011**, *480*, 520, doi:10.1038/NATURE10625.

22. Hellsten, U.; Wright, K.M.; Jenkins, J.; Shu, S.; Yuan, Y.; Wessler, S.R.; Schmutz, J.; Willis, J.H.; Rokhsar, D.S. Fine-Scale Variation in Meiotic Recombination in Mimulus Inferred from Population Shotgun Sequencing. *Proc. Natl. Acad. Sci. U. S. A.* **2013**, *110*, 19478, doi:10.1073/PNAS.1319032110.

23. Droc, G.; Larivière, D.; Guignon, V.; Yahiaoui, N.; This, D.; Garsmeur, O.; Dereeper, A.; Hamelin, C.; Argout, X.; Dufayard, J.-F.; et al. The Banana Genome Hub. *Database J. Biol. Databases Curation* **2013**, *2013*, doi:10.1093/DATABASE/BAT035.

24. Ouyang, S.; Zhu, W.; Hamilton, J.; Lin, H.; Campbell, M.; Childs, K.; Thibaud-Nissen, F.; Malek, R.L.; Lee, Y.; Zheng, L.; et al. The TIGR Rice Genome Annotation Resource: Improvements and New Features. *Nucleic Acids Res.* **2007**, *35*, D883–D887, doi:10.1093/NAR/GKL976.

25. Lovell, J.T.; Jenkins, J.; Lowry, D.B.; Mamidi, S.; Sreedasyam, A.; Weng, X.; Barry, K.; Bonnette, J.; Campitelli, B.; Daum, C.; et al. The Genomic Landscape of Molecular Responses to Natural Drought Stress in Panicum Hallii. *Nat. Commun. 2018 91* **2018**, *9*, 1–10, doi:10.1038/s41467-018-07669-x.

26. Verde, I.; Abbott, A.G.; Scalabrin, S.; Jung, S.; Shu, S.; Marroni, F.; Zhebentyayeva, T.; Dettori, M.T.; Grimwood, J.; Cattonaro, F.; et al. The High-Quality Draft Genome of Peach (Prunus Persica) Identifies Unique Patterns of Genetic Diversity, Domestication and Genome Evolution. *Nat. Genet. 2013 455* **2013**, *45*, 487–494, doi:10.1038/ng.2586.

27. Chan, A.P.; Crabtree, J.; Zhao, Q.; Lorenzi, H.; Orvis, J.; Puiu, D.; Melake-Berhan, A.; Jones, K.M.; Redman, J.; Chen, G.; et al. Draft Genome Sequence of the Oilseed Species Ricinus Communis. *Nat. Biotechnol. 2010 289* **2010**, *28*, 951–956, doi:10.1038/nbt.1674.

28. Zhou, R.; Macaya-Sanz, D.; Rodgers-Melnick, E.; Carlson, C.H.; Gouker, F.E.; Evans, L.M.; Schmutz, J.; Jenkins, J.W.; Yan, J.; Tuskan, G.A.; et al. Characterization of a Large Sex Determination Region in Salix Purpurea L. (Salicaceae). *Mol. Genet. Genomics 2018 2936* **2018**, *293*, 1437–1452, doi:10.1007/S00438-018-1473-Y.

29. Bennetzen, J.L.; Schmutz, J.; Wang, H.; Percifield, R.; Hawkins, J.; Pontaroli, A.C.; Estep, M.; Feng, L.; Vaughn, J.N.; Grimwood, J.; et al. Reference Genome Sequence of the Model Plant Setaria. *Nat. Biotechnol. 2012 306* **2012**, *30*, 555–561, doi:10.1038/nbt.2196.

30. Mamidi, S.; Healey, A.; Huang, P.; Grimwood, J.; Jenkins, J.; Barry, K.; Sreedasyam, A.; Shu, S.; Lovell, J.T.; Feldman, M.; et al. A Genome Resource for Green Millet Setaria Viridis Enables Discovery of Agronomically Valuable Loci. *Nat. Biotechnol. 2020 3810* **2020**, *38*, 1203–1210, doi:10.1038/s41587-020-0681-2.

31. The Tomato Genome Sequence Provides Insights into Fleshy Fruit Evolution. *Nature* **2012**, *485*, 635–641, doi:10.1038/NATURE11119.

32. Xu, X.; Pan, S.; Cheng, S.; Zhang, B.; Mu, D.; Ni, P.; Zhang, G.; Yang, S.; Li, R.;

Wang, J.; et al. Genome Sequence and Analysis of the Tuber Crop Potato. *Nature* **2011**, *475*, 189–195, doi:10.1038/NATURE10158.

33. McCormick, R.F.; Truong, S.K.; Sreedasyam, A.; Jenkins, J.; Shu, S.; Sims, D.; Kennedy, M.; Amirebrahimi, M.; Weers, B.D.; McKinley, B.; et al. The Sorghum Bicolor Reference Genome: Improved Assembly, Gene Annotations, a Transcriptome Atlas, and Signatures of Genome Organization. *Plant J.* **2018**, *93*, 338–354, doi:10.1111/TPJ.13781.

34. Motamayor, J.C.; Mockaitis, K.; Schmutz, J.; Haiminen, N.; Livingstone, D.; Cornejo, O.; Findley, S.D.; Zheng, P.; Utro, F.; Royaert, S.; et al. The Genome Sequence of the Most Widely Cultivated Cacao Type and Its Use to Identify Candidate Genes Regulating Pod Color. *Genome Biol.* **2013**, *14*, doi:10.1186/GB-2013-14-6-R53.

35. De Vega, J.J.; Ayling, S.; Hegarty, M.; Kudrna, D.; Goicoechea, J.L.; Ergon, Å.; Rognli, O.A.; Jones, C.; Swain, M.; Geurts, R.; et al. Red Clover (Trifolium Pratense L.) Draft Genome Provides a Platform for Trait Improvement. *Sci. Rep.* **2015**, *5*, doi:10.1038/SREP17394.

36. The International Wheat Genome Sequencing Consortium A Chromosome-Based Draft Sequence of the Hexaploid Bread Wheat (Triticum Aestivum) Genome. *Science* **2014**, *345*, doi:10.1126/SCIENCE.1251788.

37. Schnable, P.S.; Ware, D.; Fulton, R.S.; Stein, J.C.; Wei, F.; Pasternak, S.; Liang, C.; Zhang, J.; Fulton, L.; Graves, T.A.; et al. The B73 Maize Genome: Complexity, Diversity, and Dynamics. *Science* **2009**, *326*, 1112–1115, doi:10.1126/science.1178534.
